# The Rescuing Role of Aggressive Thrombosuction in Elective Coronary Angioplasty

**DOI:** 10.7759/cureus.47414

**Published:** 2023-10-21

**Authors:** Dibyasundar Mahanta, Anindya Banerjee, Abhinav Kumar, Pranjit Deb, Sindhu Rao Malla, Subhas Pramanik, Debasish Das

**Affiliations:** 1 Cardiology, SUM Hospital, Bhubaneswar, IND; 2 Cardiology, All India Institute of Medical Sciences, Bhubaneswar, IND

**Keywords:** right coronary artery, acute thrombotic occlusion, thrombosuction, aggressive, rescuing

## Abstract

Thrombosuction plays a controversial role during primary percutaneous intervention (PCI). Landmark trials have demonstrated no additional role of thrombosuction during primary percutaneous intervention towards improving mortality and outcome during primary percutaneous intervention. We describe a rare elective coronary angioplasty where only aggressive thrombosuction (almost 150-200 mL) of blood from the coronary artery established the antegrade coronary flow and saved an octogenarian from impending sudden cardiac death (SCD). The present case describes the promising role of aggressive thrombosuction even during elective coronary intervention when a large dissection ends in acute total thrombotic occlusion of a coronary artery jeopardizing the antegrade coronary perfusion.

## Introduction

During acute myocardial infarction, the coronary artery gets occluded with a long segment of occlusive thrombus jeopardizing the myocardial blood supply. During primary percutaneous intervention (PCI), there is some role of aspiration thrombectomy when the thrombus burden is too high, but clinical evidence dictates that aspiration thrombectomy does not improve mortality or outcome during primary percutaneous intervention (PCI) [1]. Although physicians prefer to do aspiration thrombectomy in patients with high thrombus burden in acute myocardial infarction, the role of aspiration thrombectomy during elective coronary angioplasty is less known. We here describe a rare case where aggressive aspiration thrombectomy (almost 150-200 mL of blood) rescued the patient and established the antegrade coronary blood flow in an octogenarian with dissection-induced acute thrombotic occlusion of the right coronary artery. Aggressive aspiration thrombectomy not only established the antegrade coronary blood flow but also improved the hemodynamics, rhythm, and saturation of the patient in a dramatic manner, whereas traditional methods to establish antegrade coronary blood flow, including dottering a semi-compliant balloon, focal balloon dilatation proximal to the totally occluded segment, intracoronary GP IIb IIIa inhibitors, and infusion of intracoronary vasodilators failed to establish the antegrade coronary blood flow. Aggressive thrombosuction still plays a role during acute thrombotic total occlusion of the coronary artery when the patient is hemodynamically unstable with arrhythmia and desaturation.

## Case presentation

**Figure 1:**
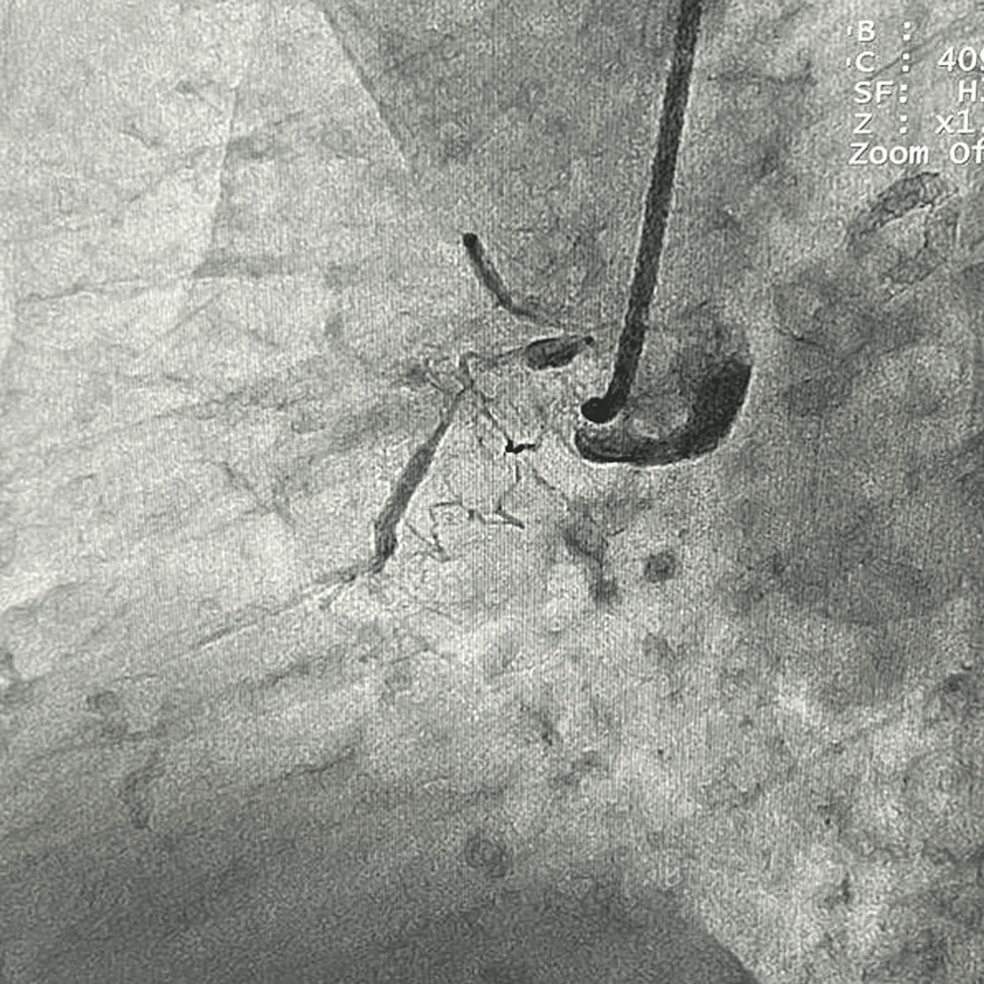
Subtotal occlusion of the right coronary artery with distal chronic total occlusion (CTO).

An 82-year-old female diabetic and hypertensive patient presented to the cardiology outpatient department with rest angina for the last three days with diaphoresis and shortness of breath New York Heart Association (NYHA) Class III without any history of palpitation, syncope, or presyncope. She had exertional angina Canadian Cardiovascular System (CCS) class II for the last five years for which she was on single antiplatelet, statin, beta-blocker, nitrate, sodium-glucose transport protein 2 (SGLT2) inhibitor, and metformin. Her blood pressure was 150/90 mmHg in the right arm supine position and her heart rate was 86 beats per minute. She had normal oxygen saturation in the room air. Baseline ECG revealed Q waves in inferior leads and echocardiography revealed no regional wall motion abnormality with preserved ejection fraction. Her quantitative troponin I was within normal range. In view of rest angina, a coronary angiogram was done which revealed subtotal occlusion of the right coronary artery with a normal left coronary system (Figure 1).

**Figure 2:**
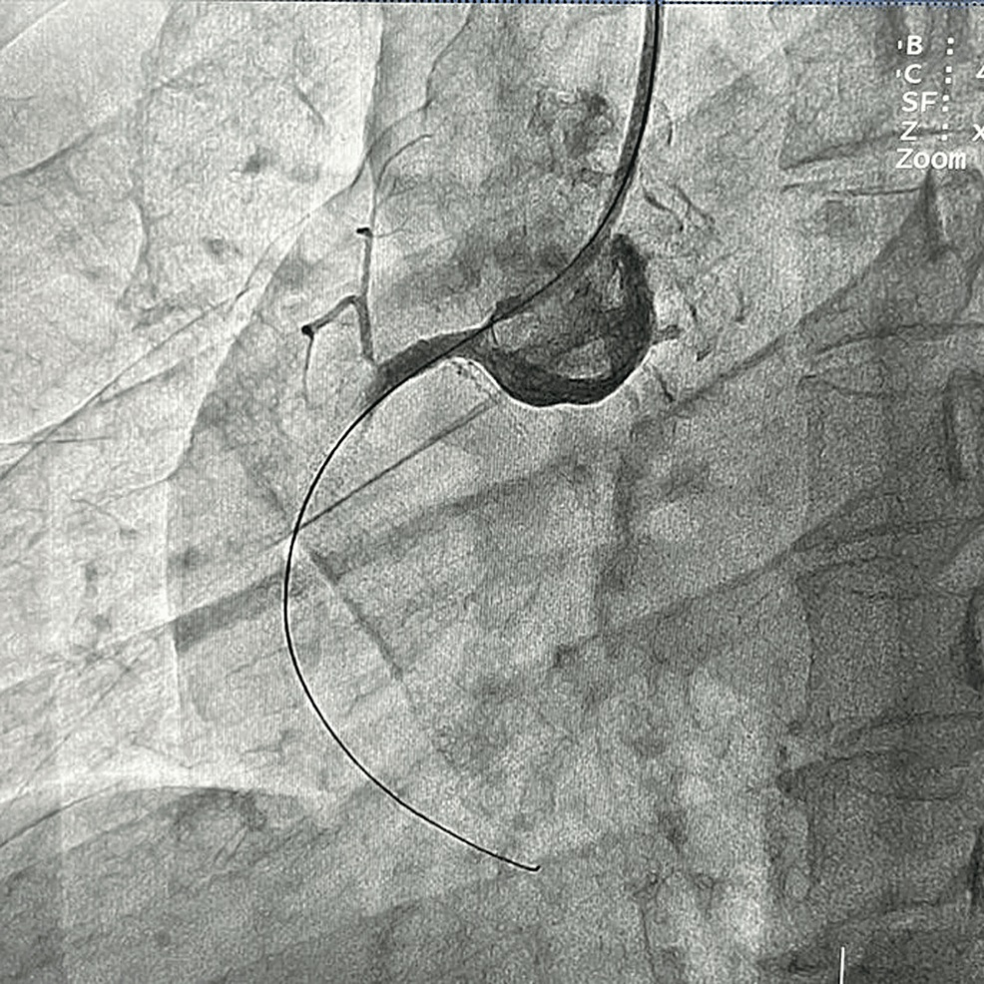
Complete thrombotic occlusion of proximal right coronary artery (RCA).

The right coronary artery was engaged with Extra-Back-Up (EBU) Guide catheter 6F 3.5 cm and the lesion in the right coronary artery was crossed with 0.014'' Fielder FC guide wire. The lesion was predilated with 1.5x10 mm and 2x10 mm semi-compliant balloons at 14-16 atm pressure. Post-balloon inflation, the lesion was stented with 3x48 mm drug-eluting stent (DES) at 14 atm pressure after which the patient was having excruciating angina, the ST segment was shooting up, developed significant bradycardia and hypotension for which the right coronary artery was injected again which revealed complete thrombotic occlusion of the proximal right coronary artery (Figure 2).

**Figure 3:**
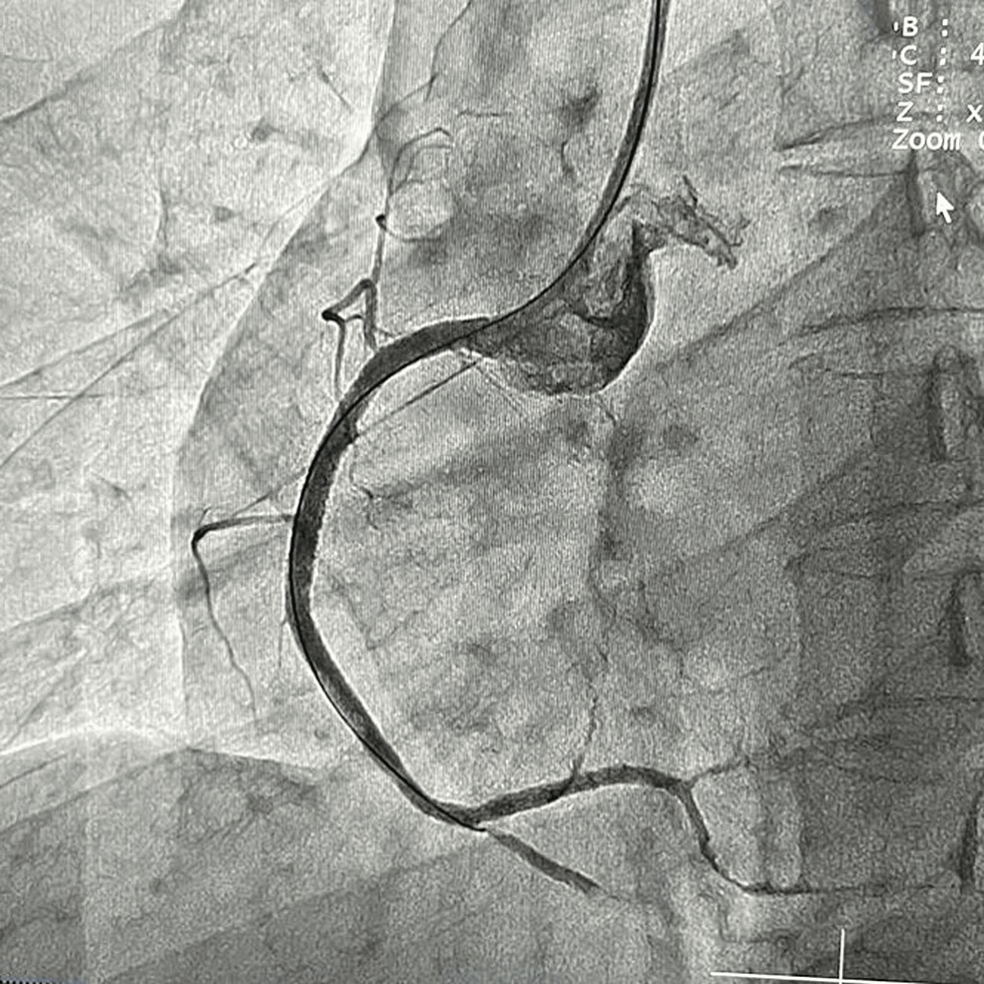
Post-aggressive thrombosuction established antegrade coronary blood flow in the right coronary artery (RCA).

We injected 3 mg of atropine, started noradrenaline infusion, and injected a further 5000 IU of unfractionated heparin. We dottered a 2x10 mm semi-compliant balloon through the occluded segment which failed to establish the coronary blood flow. We injected 10 mL of intracoronary tirofiban which also failed to establish antegrade coronary blood flow. A cocktail of intracoronary vasodilators was infused which included 200 mg of nitroglycerine and 2 mg of nicorandil which also failed to achieve distal flow. With the apprehension of proximal suboptimal stent expansion causing thrombus, the proximal stent segment was also dilated with a 3.5x10 mm semi-compliant balloon. When all the resorts to achieve antegrade flow failed, we planned to perform thrombosuction with negative pressure in a 50cc Luer lock syringe attached back to the Export thrombus aspiration catheter (Minneapolis, MN: Medtronic) aspiration thrombectomy catheter and aspirated 50-100 mL blood which did not establish antegrade flow also. Then we performed aggressive thrombosuction of the right coronary artery, almost aspirated 150-200 mL of blood after which antegrade flow was established (thrombolysis in myocardial infarction {TIMI} II flow) (Figure 3). The patient's ST elevation in ECG settled down, hemodynamics improved and saturation revived immediately. As the patient was extremely elderly, we did not continue glycoprotein (GP) IIb IIa inhibitor infusion, we put the patient on low molecular weight heparin for three days and discharged the patient in a hemodynamically stable position with isoelectric ST in inferior leads after three days. She was doing fairly well after three months of follow-up without any angina or shortness of breath. Our case is an interesting illustration of the promising and rescuing role of aggressive thrombosuction during acute thrombotic occlusion of the coronary artery even during elective coronary intervention where semi-compliant balloon-induced coronary dissection abruptly forms a large long segment clot inside the coronary artery.

## Discussion

Dissection-induced acute thrombotic total occlusion of a vessel is a rare entity during coronary intervention. Thrombosuction has been described as a potentially effective and safe method to restore coronary blood flow during primary percutaneous intervention (PCI) in acute myocardial infarction [2]. Distal thrombus migration during primary percutaneous intervention jeopardizes the myocardial blood flow extensively and is associated with a poor outcome [3,4]. Manual and vigorous aspiration of thrombus decreases the total thrombus burden in the coronary artery and decreases the risk of distal embolization, possible slow-flow, and no-reflow phenomenon. We could not establish antegrade flow by dottering a semi-compliant balloon, stent optimization in the proximal part, infusion of intracoronary tirofiban, and injecting coronary vasodilators, including nitroglycerine and nicorandil. Only vigorous thrombosuction of 150-200 mL of thrombus containing blood from the coronary artery, we could establish antegrade coronary blood flow which settled down the spiking ST elevation and restored the hemodynamics and oxygen saturation in the patient. Stone et al. demonstrated that aspiration thrombectomy prior to coronary stenting in patients with high thrombus load coronary lesions in acute myocardial infarction decreases extensive myocardial necrosis [5]. The potential advantage of thrombosuction is that it can be safely performed in hemodynamically unstable patients. As we planned for vigorous thrombosuction, we did it with negative pressure in a 50cc Luer lock syringe so that more amount of blood containing the thrombus could be aspirated. After the suction of about 100 mL of thrombus-containing blood, the antegrade flow was not established for which we planned to proceed for further thrombosuction of another 50-100 mL after which the antegrade coronary blood flow was established. Thrombosuction becomes difficult in the presence of extreme tortuosity in the vessel and when the guide catheter is not stable [6]. It is easy to perform thrombosuction in the proximal coronary vessel as compared to the distal coronary vessel because of the presence of tortuosities in the distal circulation [7]. Although intracoronary thrombectomy during primary percutaneous intervention significantly improves myocardial perfusion, our case is an interesting illustration of the role of vigorous aspiration thrombectomy in restoring antegrade blood flow during elective percutaneous coronary intervention when a large dissection induced acute total vessel thrombosis jeopardizes the myocardial blood flow and destabilize the hemodynamics and saturation with sky peaking ST-segment elevation in the surface ECG [8-10].

## Conclusions

We here emphasize the rescuing role of vigorous thrombosuction in restoring antegrade coronary blood flow during acute thrombotic total occlusion of the coronary artery during elective coronary revascularization other than primary percutaneous intervention (PCI). Vigorous thrombosuction decreases the total thrombus load in the coronary artery, restores the antegrade coronary blood flow, and improves the outcome of elective coronary angioplasty where dissection-induced complete thrombotic occlusion of a vessel can turn into a catastrophe during coronary intervention. Vigorous thrombosuction even saves lives in the presence of a high thrombus burden inside the coronary artery.
